# Polyamide 6 Nanocomposites with Inorganic Particles Modified with Three Quaternary Ammonium Salts

**DOI:** 10.3390/ma4111956

**Published:** 2011-11-02

**Authors:** Edcleide Maria Araujo, Amanda Melissa Damião Leite, Rene Anisio da Paz, Vanessa da Nóbrega Medeiros, Tomas Jeferson Alves de Melo, Hélio de Lucena Lira

**Affiliations:** Federal University of Campina Grande, Av. Aprigio Veloso, 882, Bodocongó, 58429-900, Campina Grande-PB, Brazil; E-Mails: reneanisio@yahoo.com.br (R.A.P.); vanismedeiros@gmail.com (V.N.M.); tomas@dema.ufcg.edu.br (T.J.A.M.); helio@dema.ufcg.edu.br (H.L.L)

**Keywords:** bentonite clay, nanocomposites, polyamide 6, quaternary ammonium salts

## Abstract

The purpose of this study was to obtain polyamide 6 nanocomposites with national organically modified clay with three quaternary ammonium salts. The obtained results confirm the intercalation of molecules of salt in the clay layers, and a good interaction with the polymer, showing the formation of intercalated and/or partially exfoliated structures. The nanocomposites showed similar thermal stability compared to pure polymer, and the mechanical properties presented interesting and promising results.

## 1. Introduction

Polymer composites have long been a hot topic of scientific research and industrial development, presenting a variety of applications in transportation, construction, electronics and food packaging. Nanocomposites, however, constitute a fairly new class of filled materials in which at least one dimension of the reinforcing phase is in the nanometer range [1,2]. More specifically, the use of layered silicates (clay) as reinforcement has recently gained momentum for designing polymer nanocomposites endowed with a wide array of markedly enhanced properties. In contrast with conventional fillers, the unique combination of large interfacial area and small inter-particle distance of dispersed silicates has been proved beneficial for improving polymer thermo-mechanical behavior along with barrier performance [3,4]. The research area of polymer/clay nanocomposites has attracted a great deal of attention over the last two decades. Outstanding improvements in the physical properties of polymeric matrices (e.g., stiffness, strength, heat distortion temperature, reduced permeability to gas and liquids, fire retardancy) can be expected with only few weight percents of nanofiller, therefore promising to eliminate the typical compromise between properties and processability of composite materials [5].

The first results obtained by Toyota Research [6] in the 90s on the dispersion of nanoscopic platelet silicates (montmorillonite, MMT) in a polyamide 6 matrix have induced considerable worldwide research in the field of nanocomposites consisting of a thermoplastics and clay platelet reinforcement. For very low mass fraction of platelets (less than 5%), some remarkable mechanical properties as well as thermal barrier to gases, *etc.* have led to a great industrial and academic interest [7,8,9,10]. 

Polyamide-6 (PA6) is an important group of the thermoplastic with excellent solvent resistance and good processability. However, PA6 exhibits a relatively rapid crystallization rate, which has some drawbacks such as high mold shrinkage and dimensional instability. In order to control the crystallization rate and the crystallinity, and then achieve the desired morphology and properties, a great deal of effort has been made to study the crystallization kinetics corresponding to the change of the performed properties [11]. Since the first studies on polyamide 6 (PA6)/montmorillonite (MMT) nanocomposites obtained via *in situ* intercalative polymerization [12], a wide range of polymers have been studied as hosting matrices for nanoclays resulting in a rich literature [2,3,4,5,6,7]. Nevertheless, PA6 remains a particularly successful choice for nanoclay composites and a lot of research has been devoted to the use of direct melt compounding of PA6 and, usually, smectite clays (*i.e*., MMT) as a more viable and industrially attractive preparation method than *in situ* polymerization [13,14]. 

The most frequently employed layered silicates, such as montmorillonite, are smectite-type and belong to the family of 2:1 phyllosilicates. Their crystalline structure is composed of stacked layers made of two silica tetrahedrons fused to an edge-shared octahedral sheet of aluminium or magnesium hydroxide. Pristine clays are hydrophilic and thus immiscible with most polymers, due to alkali cations residing in the interlayer to counterbalance the negative charge generated by isomorphic substitutions within the silicate sheets. However, the polymer–clay compatibility can be improved via ion-exchange reactions with organic surfactants, mainly including alkylammonium cations [15]. Extensive studies on the clay modification, especially on various types of organic surfactants have been carried out [16,17,18].

Therefore, the purpose of this study was to obtain polyamide 6 nanocomposites with national organically modified clay with three quaternary ammonium salts. The novelty of this work is the fact that we use clay of our region, which constitutes low costs and has excellent properties.

## 2. Experimental

### 2.1. Materials

Bentonite clay Brasgel PA, with CEC (cation exchange capacity) = 90 meq/100 g (method of adsorption of methylene blue) passed through ABNT sieve no. 200 (D = 74 µm), cream colored, supplied by Bentonit União Nordeste (BUN) located in Campina Grande-PB. The used polymer matrix was a polyamide 6 (Technyl C216) provided by Rhodia/SP, in the form of white pellets. Were used the three quaternary ammonium salts: Cetremide (hexadecyltrimethyl ammonium bromide), produced by Vetec/SP, Genamin (hexadecyltrimethylammonium chloride) and Dodigen (alkyl dimethyl benzyl ammonium chloride) supplied by Clariant/Brazil. 

### 2.2. Methodology

#### 2.2.1. Preparation of Organoclay

To make the clay compatible with the polymer matrix, the sodium ions present between the layers of clay were exchanged for ions of the quaternary ammonium salts (Cetremide, Genamin and Dodigen) to produce the organoclay, called OMMT-Cet, OMMT-Gen and OMMT-Dod, respectively. To obtain the organoclay (OMMT) a suitable treatment was done according to the salt, based on the CEC of clay. The detail of this procedure was reported in previous studies [19,20,21].

#### 2.2.2. Nanocomposites Preparation

The nanocomposites preparation, before any processing step, all the materials with polyamide 6 were dried in oven with circulate air at 80 °C for 1 h. Following this, these materials were kept in an oven under vacuum at 80 °C for 24 h. Nanocomposites of polyamide 6/organoclay, containing 3 wt % of clay, were melting compounded in a counterrotating twin-screw extruder (Torque Rheometer Haake) operating at 240 °C and 60 rpm. In order to assure a better dispersion of the fine clay powder in polyamide polymer, a 1:1 polyamide 6/organoclay master was previously produced in a Torque Rheometer Haake with internal mixer, at 240 °C and 60 rpm for 10 min. After mixture in extruder, samples were prepared by injection molding in a Fluidmec machine at 240 °C.

#### 2.2.3. Characterization

##### **X-Ray Fluorescence (XRF)** 

The X-ray fluorescence was conducted by semi quantitative method, of the fine powder, under Ni atmosphere, using the EDX 720 equipment of Shimadzu.

##### **X-Ray Diffraction (XRD)** 

The spacing basal of the samples was observed by X-ray diffraction (XRD) at room temperature by XRD-6000 Shimadzu diffractometer (40 kV, 30 mA) using CuKα radiation (λ = 0.154 nm) at the rate of 2° min^−1^ in the 2θ range of 2.0−30°.

##### **Thermogravimetry (TG)** 

The thermogravimetry (TG) technique was also used, by means of TGA 51 Shimadzu equipment, operating at a heating rate of 10.0 °C min^–1^, from room temperature up to 900 °C under an air atmosphere with a flow rate of 50 mL min^–1^. The sample mass was 5.0 ± 0.5 mg. The TG curves were analyzed with the aid of the TASYS software from Shimadzu.

##### **Transmission Electron Microscope (TEM)** 

The phase morphologies of the nanocomposites were observed by transmission electron microscope (TEM), Philips CM 120, operating at an acceleration voltage of 120 kV. Samples were cryogenically microtomed (−80 °C) into ultrathin sections (~30 nm thick) from films with a diamond knife using a RMC MT-7000.

##### **Mechanical Properties** 

Tensile tests were conducted using a LLoyd LR/10KN Universal Machine at room temperature and crosshead speed of 50 mm/min for the determination of tensile modulus, yield strength and elongation at yield measurements, according to the standard ASTM D638. Izod impact tests were carried out on notched specimens at room temperature using a Ceast Resil 5.5 equipment, according to the standard ASTM D256. Typically, five or more samples were used for each determination.

## 3. Results

### 3.1. X-Ray Fluorescence (XFR)

The X-ray fluorescence (XFR) technique was used to confirm the chemical composition of the clay. The results are shown in Table 1. The compositions that were observed were similar to the chemical analysis. The results also show that bromine is present for the modified clay with Cetremide and chlorine is present for the modified clay with the Dodigen and Genamin salts. These results could indicate that the washing process is not efficient.

**Table 1:** Chemical content of unmodified clay (MMT) and organoclay (OMMT) obtained by X-ray Fluorescence.

Oxides	MMT (%)	OMMT-Cet (%)	OMMT-Gen (%)	OMMT-Dod (%)
**SiO_2_**	62.85	61.72	63.55	62.69
**Al_2_O_3_**	18.02	21.19	18.66	26.77
**Fe_2_O_3_**	11.25	9.88	11.91	7.30
**MgO**	2.06	2.91	1.60	-
**CaO**	1.78	-	0.96	0.90
**TiO_2_**	1.22	1.00	1.28	0.77
**Na_2_O**	1.12	-	-	-
**K_2_O**	0.85	0.58	0.84	0.68
**Cl**	-	-	0.76	0.33
**Br**	-	1.07	-	-
**others**	0,46	0.63	0.44	0.56

### 3.2. X-Ray Diffraction (XRD)

**Figure 1:**
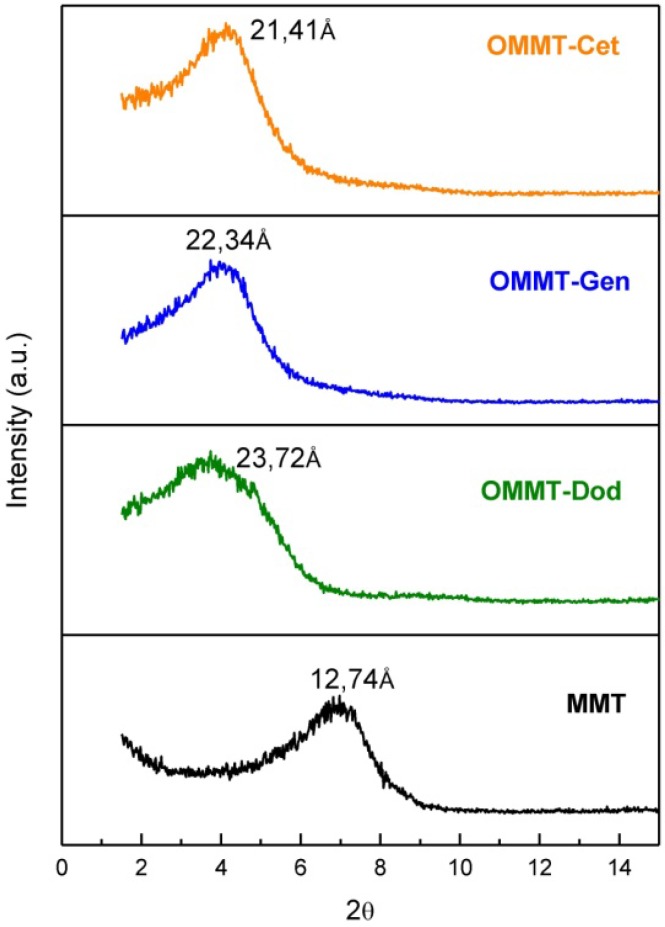
X-ray diffraction (XRD) patterns of the clay without treatment (MMT) and clay treated with the Dodigen (OMMT-Dod), Genamin (OMMT-Gen) and Cetremide (OMMT-Cet) salts.

The XRD were made to the clay without treatment (MMT) and clay treated with three different salts, Dodigen, Genamin and Cetremide. Figure 1 shows that there was a shift of the 2θ angle of 6.97° (MMT, d_001_ = 12.74 Å) to 3.74° (OMMT-Dod, d_001_ = 23.72 Å) to 4.01° (OMMT-Gen, d_001_ = 22.34 Å) and 4.15° (OMMT-Cet, d_001_ = 21.41 Å). The shift to smaller angles and, consequently, increasing the basal spacing, indicates the intercalation of molecules of the salt among the layers of clay. 

Considering that the length of C-C bond is 1.54 Ǻ and that Genamin (hexadecyltrimethylammonium chloride) and Cetremide (hexadecyltrimethyl ammonium bromide) each have 16C, the expected length of the chain of these compounds is close to 24.64 Ǻ. In this study, the basal spacing of the MMT changes from 12.74 Ǻ to 22.34 Ǻ and 21.41 Ǻ when treated with Genamin and Cetremide, respectively. This suggests that the molecules of quaternary ammonium salt probably present a lie flatly and interact relatively strongly with the montmorillonite packet surface forming a monolayer in the montmorillonite interlayer space. This is in agreement with Kozaka, and Domka [22] who studied the adsorption of the quaternary ammonium salts on montmorillonite and found a value of 15.57 Ǻ for 12C. Also Kwolek *et al*. [23], who, when studying the adsorption isotherms of homologous alkyldimethylbenzylammonium bromides on sodium montmorillonite, found that the organoclay intercalated with 12C, presented basal spacing of 17.969 Å and suggested that the cations also lie flatly and interact relatively strongly with the montmorillonite packet surface. Dodigen presented basal spacing of 23.72 Ǻ and its molecule presents one chain with 12 or 14C and one benzyl group. In this case, the steric properties of the structure of the compounds probably form a monolayer of ammonium chloride or a disordered bilayered structure. 

**Figure 2:**
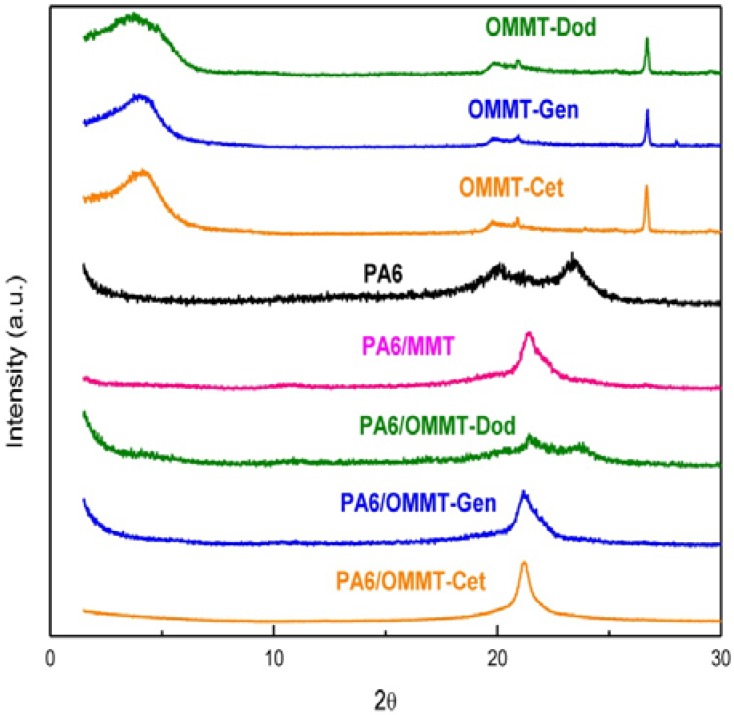
XRD patterns of organoclay, polyamide 6 (PA6) and nanocomposites of PA6/clay.

Figure 2 shows the results of X-ray diffraction for organoclay and the nanocomposites of polyamide 6 (PA 6). The nanocomposites were analyzed with films made in a twin screw extruder, with the content of 3 wt % clay. As seen in Figure 1, the peak is present for the clays with the salts and it disappears when there is the incorporation of clay in polymer matrix. These results apparently show that the nanocomposites have exfoliated structure in accordance with the literature [3].

According to Khanna and Kuhn [24], polyamide 6 can take two crystallographic forms, α monoclinic and γ pseudo-hexagonal. In the α form, the hydrogen bonds are formed between antiparallel chains, and γ form between parallel chains by hydrogen bonds, causing the twist of the molecular chains in zig-zag planes. As a result of this phenomenon, the crystal density and heat of fusion of γ form, where interactions between the chains are weaker, are less than the α form.The α form can be identified in XRD pattern of X-ray diffraction peak at 2θ approximately 24°, the crystalline γ form is shown in Figure 2 as a peak between 21 and 22°. As seen, the introduction of treated clay changes the shape of these peaks, probably by changing the crystallinity of PA6.

### 3.3. Transmission Electron Microscope (TEM)

**Figure 3:**
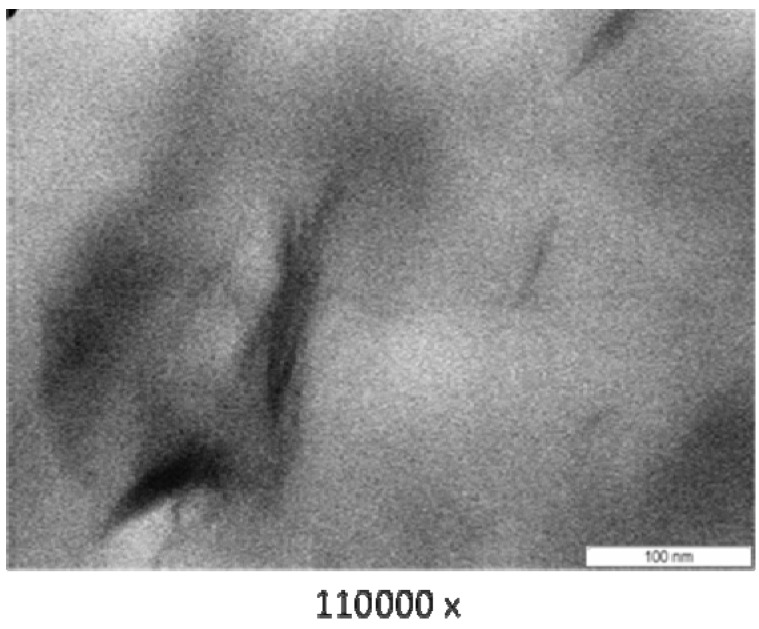
Transmission electron microscope (TEM) photomicrographs for the film nanocomposite PA6/MMT.

**Figure 4:**
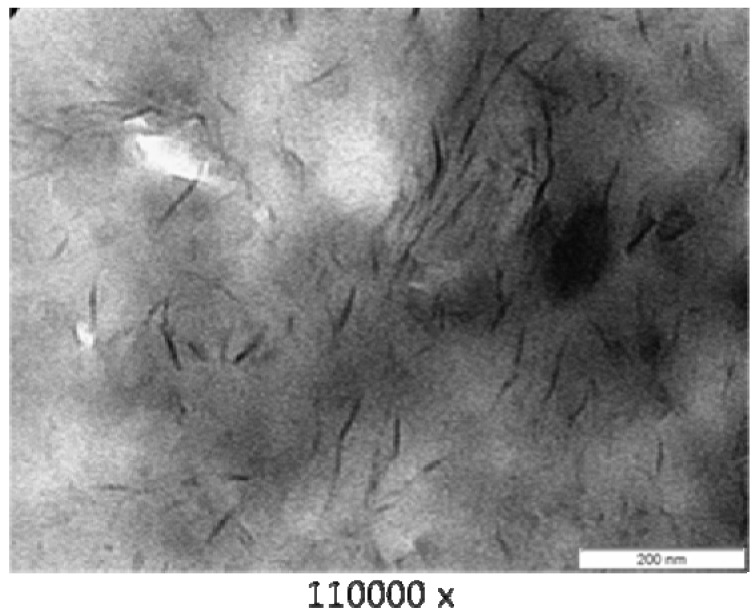
TEM photomicrographs for the film nanocomposite PA6/OMMT-Dod.

**Figure 5:**
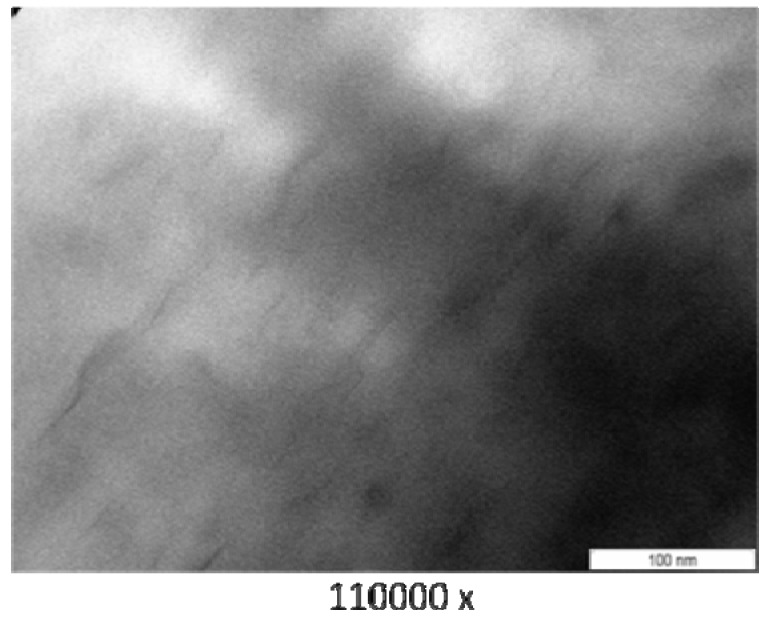
TEM photomicrographs for the film nanocomposite PA6/OMMT-Gen.

**Figure 6:**
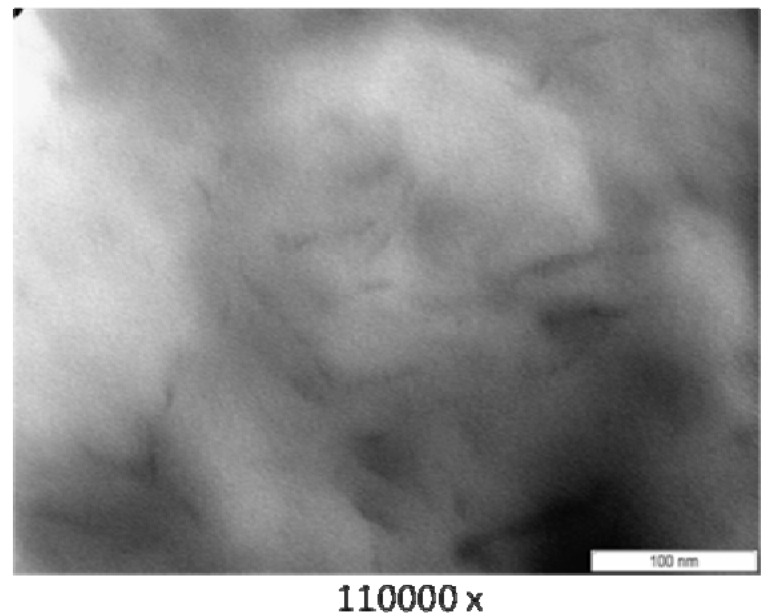
TEM photomicrographs for the film nanocomposite PA6/OMMT-Cet.

Figure 3 (PA6/MMT), Figure 4 (PA6/OMMT-Dod), Figure 5 (PA6/OMMT-Gen) and Figure 6(PA 6/OMMT-Cet) show the photomicrographs of the systems of polyamide 6 with 3% by weight of organoclay.

Photomicrographs of the systems PA6/MMT (Figure 3), PA 6/OMMT-Gen (Figure 5) and PA 6/OMMT-Cet (Figure 6) show a partially exfoliated morphology, composed of layers of clay and some clusters dispersed in the polymer matrix, and the system PA 6/MMT shows clusters of larger sizes.

The system PA 6/OMMT-Dod (Figure 4) shows an exfoliated morphology with the presence of layers of clay uniformly distributed, also with some clusters.

In this work, we observed that, for the studied quaternary ammonium salts, the nanocomposites exhibited a structure with a predominance of exfoliation, and can confirm the results previously obtained and displayed X-ray diffraction (Figure 2), thus requiring the use these techniques for characterization and interpretation of the type of obtained nanocomposite.

### 3.4. Thermogravimetry (TG)

**Figure 7:**
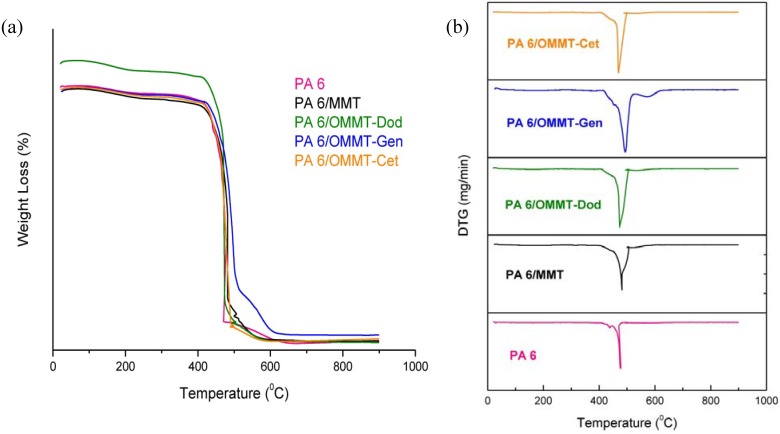
TG (**a**) and DTG (**b**) curves of polyamide 6 and its nanocomposites.

Figure 7 shows the TG and DTG curves for the polyamide and its nanocomposites in air atmosphere. It can be seen that the polyamide 6 and its nanocomposites showed similar behavior, since there was a mass loss ranging from approximately 400 to 520 °C, with a maximum in the range of 478 °C on the decomposition of the nanocomposite, with the exception of the nanocomposite that used the modified clay with salt Genamin, with a maximum at 490 °C and 620 °C, the final decomposition of the nanocomposite. Apparently, there were no major changes in the thermal behavior between polyamide 6 and its nanocomposites, which is interesting because of the thermal stability of these materials in the presence of modified clay. The nanocomposite that used the clay with Genamin salt showed a maximum temperature of decomposition of 490 °C, which is higher than the others.

### 3.5. Mechanical Properties

Table 2 shows yield strength, tensile modulus, elongation at yield and Izod impact strength of pure PA6 and its nanocomposites. It was seen that the nanocomposites showed improved mechanical properties compared to pure polyamide, in the others words, the clay acted as a nucleating agent, increasing the stiffness of the material. It was also observed that there was a reduction in impact strength for the systems of PA6/MMT, PA6/OMMT-Dod and PA6/OMMT-Gen in relation to pure PA6 and an increase of approximately 10% of the PA6/OMMT-Cet in comparison the pure PA. Although, it used only 3% of clay, an enhancement in mechanical properties was observed. Studies are being made to verify the influence of the content of clay in the mechanical properties of the nanocomposites.

**Table 2:** Mechanical properties of PA 6 and its nanocomposites.

Specimens	Tensile Modulus (GPa)	Yield Strength (MPa)	Elongation at Yield (%)	Izod Impact Strength (J/m)
**Pure PA6**	2.15 ± 0.09	64.97 ± 0.56	16.06 ± 0.65	45.78 ± 1.6
**PA6/MMT**	2.40 ± 0.04	68.52 ± 0.90	11.54 ± 0.41	44.33 ± 1.0
**PA6/OMMT-Dod**	2.71 ± 0.13	71.07 ± 1.1	12.66 ± 0.07	28.17 ± 1.41
**PA6/OMMT-Gen**	2.48 ± 0.24	71.18 ± 0.28	11.03 ± 0.71	34.65 ± 1.91
**PA6/OMMT-Cet**	2.42 ± 0.13	69.70 ± 0.49	12.98 ± 0.44	48.98 ± 4.27

## 4. Conclusions

The effect of organoclay with three types of quaternary ammonium salts in the properties of PA6/Brazilian clay nanocomposites was studied. The results of XRD indicated that all the quaternary ammonium salts were intercalated among two basal planes of MMT, leading to an expansion of the interlayer spacing and the PA6 nanocomposites with exfoliated structure. These results were confirmed by TEM. The results of thermogravimetry indicated similar thermal behavior between polyamide 6 and its nanocomposites. This can be attributed to interactions between the set of polymer chains and the organic compounds of modified clays. The reinforcing effect of clay in the polymer was confirmed by the increased rigidity of the system. In general, the data showed quaternary ammonium salts to be efficient in the organophilization of clay and suggested that the Brazilian clay can be used as a nanoparticle in PA6 nanocomposites.
